# Surface Physicochemistry and Ionic Strength Affects eDNA’s Role in Bacterial Adhesion to Abiotic Surfaces

**DOI:** 10.1371/journal.pone.0105033

**Published:** 2014-08-14

**Authors:** Viduthalai R. Regina, Arcot R. Lokanathan, Jakub J. Modrzyński, Duncan S. Sutherland, Rikke L. Meyer

**Affiliations:** 1 Interdisciplinary Nanoscience Center (iNANO), Aarhus C, Denmark; 2 Department of Bioscience, Aarhus University, Aarhus C, Denmark; INRA Clermont-Ferrand Research Center, France

## Abstract

Extracellular DNA (eDNA) is an important structural component of biofilms formed by many bacteria, but few reports have focused on its role in initial cell adhesion. The aim of this study was to investigate the role of eDNA in bacterial adhesion to abiotic surfaces, and determine to which extent eDNA-mediated adhesion depends on the physicochemical properties of the surface and surrounding liquid. We investigated eDNA alteration of cell surface hydrophobicity and zeta potential, and subsequently quantified the effect of eDNA on the adhesion of *Staphylococcus xylosus* to glass surfaces functionalised with different chemistries resulting in variable hydrophobicity and charge. Cell adhesion experiments were carried out at three different ionic strengths. Removal of eDNA from *S. xylosus* cells by DNase treatment did not alter the zeta potential, but rendered the cells more hydrophilic. DNase treatment impaired adhesion of cells to glass surfaces, but the adhesive properties of *S. xylosus* were regained within 30 minutes if DNase was not continuously present, implying a continuous release of eDNA in the culture. Removal of eDNA lowered the adhesion of *S. xylosus* to all surfaces chemistries tested, but not at all ionic strengths. No effect was seen on glass surfaces and carboxyl-functionalised surfaces at high ionic strength, and a reverse effect occurred on amine-functionalised surfaces at low ionic strength. However, eDNA promoted adhesion of cells to hydrophobic surfaces irrespective of the ionic strength. The adhesive properties of eDNA in mediating initial adhesion of *S. xylosus* is thus highly versatile, but also dependent on the physicochemical properties of the surface and ionic strength of the surrounding medium.

## Introduction

Bacteria adhere to almost all kinds of surfaces, enabling biofilm formation [1]. Biofilms can cause serious health problems as well as economic losses in many industries, such as the oil and gas industry where biofilms cause corrosion, and the food industry where spoilage, and contamination leading to food-borne illnesses are the main concern [2]–[10]. While antimicrobial surfaces releasing toxins are the traditional approach to antifouling solutions, development of non-toxic antifouling surfaces that intercept cell adhesion and biofilm formation rather than cell viability are gaining more interest [11]. However, development of such antifouling strategies relies on detailed understanding of the mechanisms behind bacterial adhesion and the subsequent establishment of a biofilm. Bacterial adhesion is a complex process involving long range Lifshitz van der Waals and electrostatic forces, as wells as short range acid-base interactions [12], [13]. The physico-chemistry of the substrate surface and the bacterial surface decides the extents of these interactions and hence the adhesion of bacteria. However, bacterial adhesion cannot always be predicted by the average physicochemical properties of the cell surface. Specific extracellular components that might evade the interaction force barrier formed between the approaching surfaces are often the primary facilitator for adhesion, even if they do not contribute substantially to the average physicochemical surface properties of the cell [14]–[16]. Such extracellular components include carbohydrate polymers, single and fibrillar proteins, and extracellular DNA (eDNA) [17].

The active role of eDNA in biofilm formation was discovered by Whitchurch *et al.* [18], who showed that removal of eDNA by DNase treatment could dissolve young *Pseudomonas aeruginosa* biofilms and prevent biofilm formation on abiotic surfaces. Since then, the involvement of eDNA in biofilm formation has been studied in many different bacteria across several phyla, and an image of eDNA as a universal adhesin is emerging. But what makes eDNA adhesive? Only few studies have attempted to address how eDNA promotes bacterial adhesion to abiotic surfaces. Das *et al*. [19] studied adhesion of *Streptococcus mutans*, and used AFM force spectroscopy to investigate how eDNA increased adhesion forces of bacterial cells to hydrophobic and hydrophilic surfaces at varying ionic strength (*I*). They found that presence of eDNA on the cell surface increased the adhesion strength to both hydrophilic and hydrophobic surfaces. The overall adhesion strength in the presence of eDNA was higher to hydrophobic surfaces than to hydrophilic surfaces, and the effect was more pronounced at high ionic strength. Furthermore, AFM retraction-force distance curves revealed that eDNA-mediated acid-base interactions were more pronounced in the interaction with hydrophilic surfaces, and that these interactions were high in low ionic strength. Ionic strength can affect the electrostatic properties of surfaces and also influence the conformation of biopolymers such as DNA. The ability of eDNA to function as an adhesive thus seems to occur within certain boundaries, defined by the physico-chemical properties of the substrate and the surrounding environment.

To get a better understanding of eDNA’s versatility as a universal bacterial adhesin, we investigated how surface chemistry and ionic strength affects eDNA’s role in bacterial adhesion. We used *Staphylococcus xylosus* as a model organism. *S. xylosus* are Gram positive, biofilm forming, bacteria that have frequently been isolated from food and food related environments [20], [21]. These bacteria can produce aroma [22] and enterotoxins that qualify them as potential contaminants in food processing industries [23]. Furthermore, some strains of *S. xylosus* have been reported to carry antibiotic resistance genes [24] which is a major concern in the emerging threat of antibiotic resistance among bacteria. These characteristics makes *S. xylosus* an attractive candidate to study the factors influencing biofilm formation *in* *vitro*. In this study we quantified initial bacterial adhesion to a range of chemically modified surfaces, representing surfaces with varying charge and hydrophobicity, and repeated experiments at three different ionic strengths. eDNA is critical for the adhesion of bacteria to both hydrophilic and hydrophobic surfaces. Electrostatic interactions, modulated by surface chemistry, depended on the ionic strength in facilitating eDNA mediated adhesion to hydrophilic surfaces. The amount of eDNA released by the cells, their ability to use eDNA and/or a combination of other adhesins for adhesin will be different for different types of bacteria. Therefore, the observed effects on eDNA mediated adhesion of *S. xylosus* may not be generalized to all biofilm forming bacteria. However, the findings presented in this study can be used to determine the range of physico-chemical characteristics wherein eDNA can influence adhesion of bacteria to abiotic surfaces.

## Results and Discussion

### eDNA is critical for adhesion to glass

**Figure 1:**
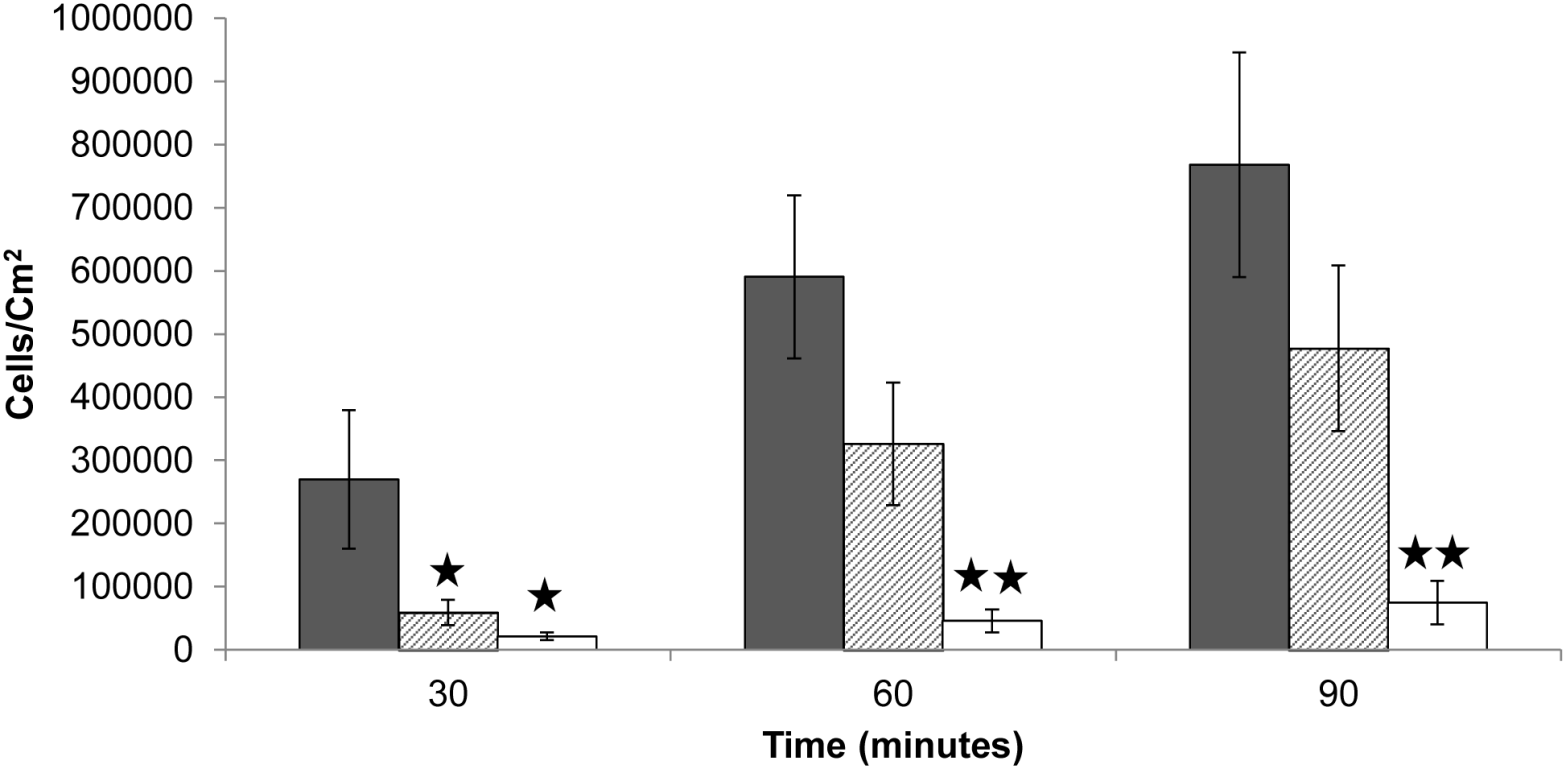
Effect of DNase on the adhesion of *S. xylosus* to glass surface in flow cell. Black bars indicate untreated cells. Crossed bars indicate cells treated with DNase (50 µg/ml), washed and resuspended in PBS. White bars indicate cells resuspended in PBS containing DNase (50 µg/ml). Asterisk indicates statistically significant differences between samples with and without eDNA (t-test, *p<0.05, **p<0.01).

The presence of eDNA was critical for adhesion of *S. xylosus* to glass surfaces, as removal of eDNA by DNase treatment almost fully impaired adhesion of cells (Figure 1). Interestingly, the cells quickly regained their adhesive properties if the DNase was removed from the cell suspension before the onset of the adhesion experiment. In this case, adhesion of *S. xylosus* to glass was only delayed by approximately 30 min, after which the adhesion rate was not significantly different from the untreated control (Figure 1) (Two-way ANOVA; *F* = 0.03, *p* = 0.87). Only if DNase was continuously present, adhesion was impaired for the entire duration of the experiment (Figure 1). Autolysis caused by the activity of the major autolysin AltE is responsible for the eDNA in biofilms formed by other *Staphylococcus* species [25]–[27], and the same mechanism may be present here. The short time needed for the cells to regain their adhesive properties after removing the DNase from the cell suspension suggests that eDNA was continuously released in the culture, and that the amount of eDNA needed to facilitate cell adhesion is very low. We could not quantify the amount of eDNA adsorbed to the cells, but the eDNA concentration in the supernatant of the cell suspension was 110.2±11.5 ng/ml for the untreated cells, whereas DNase treated cells contained 15.5±0.2 ng/ml for bacterial suspensions of 1.3×10^8^ cells.

### eDNA increases cell surface hydrophobicity but does not affect surface charge

To better understand the adhesive properties of cells with eDNA, we investigated the average physicochemical properties of untreated and DNase-treated cells by measuring the cell surface hydrophobicity and charge as approximated by the water contact angle and zeta potential, which is a measure of potential at the diffuse layer of ions formed around particles in aqueous medium. To account for the effect of ionic strength on zeta potential, measurements were done in PBS and 10 times diluted PBS. The zeta potential of the cells was not affected by DNase treatment (Table 1). Cells suspended in 10 times diluted PBS (low ionic strength compared to PBS) had more negative zeta potential, but remained unaffected by DNase treatment (Table 1). Hence, the adsorption of eDNA to the cell surface does not appear to alter the average surface charge of *S. xylosus.*

**Table 1:** Cell surface properties with and without eDNA.

*S. xylosus* cells	Water Contact angle (°)	Zeta potential (mV)	
		10 mM PBS	1 mM PBS
with eDNA	46.7±3.7	–15.1±0.9	–34.0±2.4
without eDNA	*33.5±2.6	–15.1±0.9	–34.4±1.3

Water contact angle and zeta potential measurements of *S. xylosus* cells with and without eDNA.

*indicates statistically significant difference (t-test, p<0.05).

Das *et al.* [28] did find a more negative zeta potential after adding DNA to *S. aureus, S. epidermidis* and *P. aureginosa* cultures, but only at relatively high concentrations (>4–6×10^−9^ µg DNA per bacterium). As we have alluded before, the concentration of naturally occurring eDNA in our cell suspensions was very low (approximately 110 ng/ml, which corresponds to 8.5×10^−7^ ng per cell), and the amount of eDNA present may not have been sufficient to cause a detectable difference in cell surface charge. Another explanation could be that nucleotides or short strands of eDNA present after DNase treatment remain associated with the cell surface, and thus could contribute to the zeta potential. If eDNA in this form is unable to promote cell adhesion, DNase treatment could affect the adhesive properties of the cells without affecting their surface charge.

The water contact angle was significantly lower after DNase treatment, indicating that cells with eDNA are more hydrophobic (Table 1). The same phenomenon was observed by Das *et al.* for other staphylococci [28], [29] who showed that *S. epidermidis* cells became less hydrophobic when lacking eDNA due to either DNase treatment or deletion of the *altE* gene that facilitates eDNA release through autolysis. They also showed that adding increasing amounts of DNA to the culture resulted in increasing cell surface hydrophobicity. DNA in itself is not hydrophobic, and it is not yet understood why removal of eDNA makes the cell surface less hydrophobic. However, it has been suggested that DNA is associated with other components on the cell exterior [30]–[36], and these might contribute to the hydrophobic cell surface properties. One study simply showed that the adhesive properties of eDNA-free *Nisseria meningiditis* could be restored by addition of genomic DNA from crude extracts, but not from purified DNA [33], indicating a role of a non-DNA component in eDNA-mediated adhesion. More specific knowledge was obtained from a similar experiment on *Listeria monocytogenes* showing that N-acetyl glucosamine as well as DNA was needed to restore the adhesive properties of eDNA-free cells [32]. Several studies have sought to determine if proteins are involved in eDNA-mediated adhesion. For example, DNA-binding proteins, such as the pilin protein of Type IV pili [37], play a part in eDNA’s role in biofilm formation by e.g. *Acidovorax temperans* [38] and *P. aeruginosa* [39]. Furthermore, Das *et al.* [35] showed that pyocyanin, a phenazine molecule produced by *P. aeruginosa*, affects cell surface properties and aggregation by facilitating the binding of eDNA binding to the cell surface [35]. Whether these are isolated examples or represent a general picture of eDNA being a partner in a multi-adhesin system is yet to be revealed, and the attention should be aimed at small molecules as well as macromolecules in the search for other extracellular components partnering with eDNA in mediating biofilm formation.

### eDNA’s effect on adhesion depends on surface chemistry and ionic strength

Bacterial adhesion is dictated by long and short range forces between the cell and the surface [13], [40]. Liefshitz van der Waals and electric double layer forces are long range forces (several nanometers), where the former is attractive and the latter can be both attractive and repulsive. In contrast, Lewis acid-base interactions operate at short range [12]. The physico-chemistry defines the extent of these forces and thereby decides the interaction between approaching surfaces, but the interactions are also influenced by the properties of the surrounding liquid. Ionic strength of the liquid affects the thickness of the electric double layer, and thereby the electrostatic interactions [41]. The thickness of the electric double layer (the Debye length (δ)) is estimated by equation (1) where *ε* is the permittivity (determined from *ε_r_ε_0_*, where ε_r_ is the relative permittivity at temperature, T = 300 K and ε_0_ is the vacuum permittivity), *k* is the Boltzmann constant, *e* is the electron charge, *c* is the concentration, and *z* is the charge of the electrolyte.
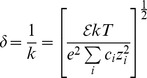
(1)


The Debye length at low, intermediate and high ionic strengths were 1.75 nm, 0.49 nm and 0.25 nm respectively.

The conformation of biopolymers, such as eDNA, can also be influenced by ionic strength and ionic composition of the surrounding medium [42], and we therefore hypothesized that the ionic strength of the surrounding media as well as the physico-chemical properties of the abiotic surface will affect eDNAs role in bacterial adhesion. In order to test this hypothesis, we quantified the adhesion of bacteria to glass surfaces with different surface chemistries in the presence or absence of DNase. We modified glass cover slips to obtain an array of surfaces with highly different chemistries, representing different hydrophobicity and charge, and submerged the cover slips in bacterial suspensions in 12 well plates under continuous shaking before rinsing and quantifying the adhered bacteria.

XPS analyses confirmed the presence of the functional groups on each surface (Figure S1). The Piranha treated and carboxyl functionalised surfaces were negatively charged with zeta potentials of –55.7±2.5 mV and –70.4±5.9 mV respectively, and highly hydrophilic with water contact angles of only 4.6±0.8° and 5.5±2.3°, respectively. Amine functionalised surfaces were positively charged, (84.6±4.9 mV) and slightly less hydrophilic with a water contact angle of 30.3±4.8°, and the fluoro silane functionalised surfaces were slightly negatively charged (–13.7±0.9 mV) and were the only hydrophobic surfaces tested, having a water contact angle of 73±3.1°.

**Figure 2:**
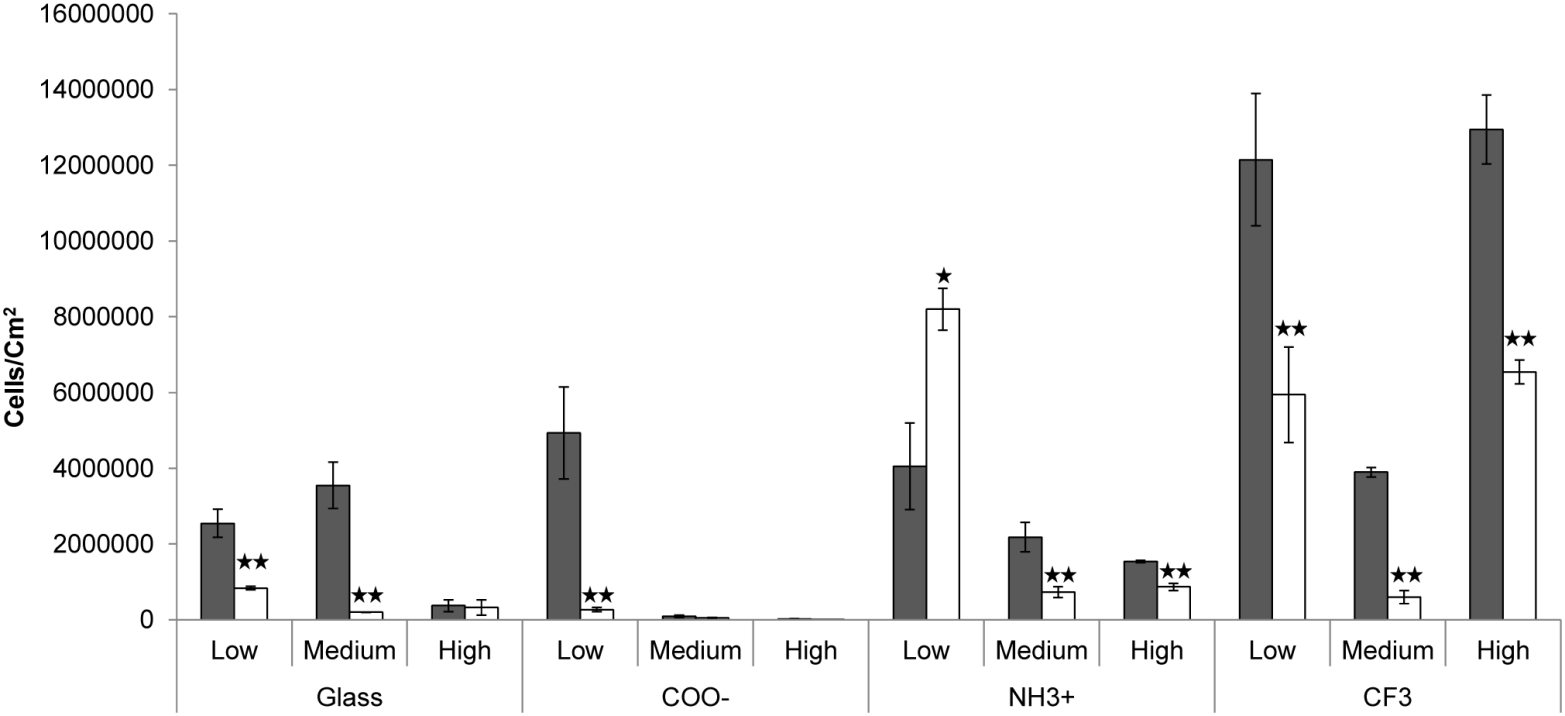
Effect of ionic strength and surface chemistries on eDNA mediated adhesion of *S. xylosus*. Black bars indicate untreated cells. White bars indicate cells treated with DNase. Experiments were carried out at low (I = 0.015 M), medium (I = 0.19 M) and high (I = 0.70 M) ionic strength. Values are average of 3 replicates (error bars  =  S.D.) Asterisk indicates statistically significant differences between samples with and without eDNA (t-test, *p<0.05, **p<0.01).

eDNA stimulated adhesion to all surfaces, but not to the same extent, and not at all ionic strengths. Very little adhesion occurred to the hydrophilic and negatively charged Piranha treated and carboxyl functionalised glass surfaces in the absence of eDNA (Figure 2). However, the stimulating effect of eDNA was only evident at low and intermediate *I*. Das *et al.* [28] showed that eDNA mediates bacterial adhesion through short range Lewis acid-base interactions and the same authors argued that these forces are more pronounced in eDNA-mediated adhesion at low *I* and toward hydrophilic as opposed to hydrophobic surfaces [19]. We speculate that loops of eDNA must extend from the cell surface and protrude the electric double layer to facilitate adhesion. Indeed Das *et al.* showed that eDNA extended approximately 400 nm from the cell surface of *Streptococcus mutans* cells [28], and the thickness of the electric double layer determine here was only a few nm. Furthermore, DLS measurements have confirmed that eDNA only increases the hydrodynamic radius of bacteria at low *I* [19], supporting our hypothesis that eDNA must extend from the bacterial cell surface to mediate adhesion to hydrophilic surfaces.

At the intermediate ionic strength, eDNA stimulated adhesion to Piranha treated but not carboxyl functionalised glass surfaces. The negatively charged hydrophilic glass surfaces are known to contain localized positive charges [43], which are important for adhesion mediated by long range electrostatic interactions [15]. While these are masked by the thicker electric double layer and the overall negative charge at low electrolyte concentrations, they may become visible at high electrolyte concentrations. Such positive charges may not be accessible for the eDNA on carboxyl functionalized surfaces as they were modified by self-assembled monolayers of (Triethoxysilyl) propylsuccinic anhydride, but the availability of these charges on the Piranha treated glass may have contributed to eDNA-mediated adhesion despite the increase in *I.*

eDNA promoted adhesion of cells to positively charged aminated surfaces in medium and high *I* (Figure 2), but surprisingly, it had the opposite effect at low *I*. Electrostatic interactions are more predominant at low *I*, and we had therefore expected that the negatively charged eDNA would promote adhesion to amine-functionalised under these conditions. We did not investigate the underlying mechanism is behind this result further, but it is likely caused by the role of Mg^2+^ ions in the absence of other salts. Divalent cations like Mg^2+^ interact with the phosphate backbone of DNA and provide charge neutralization [44], which allows DNA to adsorb to negatively charged surfaces [45]. Mg^2+^ is therefore commonly used to facilitate DNA adsorption to negatively charged mica surfaces, e.g. for atomic force microscopy [46], and conversely, EDTA is used to chelate Mg^2+^ in order to keep DNA in suspension for molecular biology research. The charge neutralization of Mg^2+^ is approximately 100 fold more effective compared to monovalent ions [45], and we therefore expect that charge neutralization of eDNA on the bacterial cell surface is most significant in the low *I* incubation, where Mg^2+^ does not compete with Na^+^ and K^+^ for the interaction with the phosphate groups of the DNA backbone. Indeed, Nguyen et al. (2007) showed that the presence of Mg^2+^ enhanced DNA adsorption to natural organic matter, but to a lesser extent in the presence of 7 mM NaCl [47]. The NaCl concentration in 10 mM PBS is 149 mM, hence the effect of Mg^2+^ on eDNA-mediated bacterial adhesion will be much less in PBS. The charge neutralization of DNA by Mg^2+^ in the absence of other ions can explain that eDNA lowered bacterial adhesion to the positively charged amine-functionalised surfaces and promoted adhesion to the negatively charged carboxyl-functionalised surfaces at low *I*. The presence of Mg^2+^ was required for the activity of DNase, and we could therefore not perform control experiments in the absence of Mg^2+^ to test the hypothesis.

In contrast to the hydrophilic surfaces, eDNA promoted adhesion of *S. xylosus* to the neutrally charged hydrophobic surfaces irrespective of the *I* of the surrounding liquid (Figure 2). Hydrophobic surfaces in general cannot contribute to acid-base interactions due to the lack of polar groups. *S. xylosus* cells were more hydrophobic in the presence of eDNA (Table 1), and when hydrophobic surfaces approach each other, Lifshitz-van der Waals forces increase due to the removal of interfacial water [48]. This increase in Lifshitz-van der Waals interaction facilitates faster approach of cells towards the surface, hence promoting adhesion. Lifshitz-van der Waals forces can be overcome by electrostatic forces at low ionic strength [49], however the hydrophobic surface, functionalized with fluorine end groups (-CF_3_), cannot contribute to such forces. Das *et al*. [19] also found that eDNA creates a favorable conditions for bacterial adhesion to hydrophobic surfaces, and demonstrated this both theoretically through thermodynamic calculations, and experimentally by AFM adhesion force spectroscopy measurements [19], [29]. DNA is an amphipathic molecule having a hydrophilic backbone and hydrophobic bases in the core. These nitrogenous bases might act as structures that participate in the hydrophobic interactions. This reinforces the idea that eDNA on the cell surface may be partially or completely single stranded, allowing the hydrophobic bases to get exposed. However, further investigation is needed to strengthen this hypothesis.

Collectively, our data and previous studies suggest that eDNA can participate both in short-range acid-base interactions and long-range electrostatic and Lifshitz-van der Waals interactions, and eDNA is thus able to stimulate bacterial adhesion to a wide range of surface chemistries. With eDNA being used in biofilm formation by a wide range of bacteria from several different phyla, an image of eDNA as a universal adhesin is emerging. In nature, eDNA-mediated biofilm formation is believed to occur at low ionic strength in aquatic environments, and as most surfaces in nature are negatively charged [50]–[52], our data support this hypothesis. The effect of ionic strength on the conformation of eDNA might be the key feature preventing its role in mediating the adhesion at high ionic strength. However, the ability of eDNA to aid adhesion to hydrophobic surfaces under any conditions raises new questions about the possible role of eDNA in e.g. cell aggregation or attachment to debris in marine environments. The versatility of eDNA as an adhesive may yet reveal new situations where eDNA-mediated adhesion increases survival of bacteria. Furthermore, the emerging image of eDNA as an important adhesin for many bacteria across several phyla points to eDNA as a potential global target in biofilm prevention. Understanding the mechanism by which eDNA mediates bacterial adhesion is therefore an fundamental basis for development of a new generation of antifouling surfaces or cleaning regimes that enzymatically degrade DNA or interfere with eDNA’s adhesive properties.

## Materials and Methods

### Preparation of bacteria

A *Staphylococcus xylosus* (DSM 20266, DSMZ, Braunschweig, Germany) starter culture was inoculated from agar plates and grown in 3 ml of 1% TSB medium in 50 ml conical bottom tube by incubating overnight with shaking at 30°C. One ml from this culture was then used as inoculum for 100 ml of 1% TSB medium, which was incubated overnight, harvested by centrifugation (5 min at 3000×g), washed twice and resuspended in phosphate buffered saline (PBS, pH 7.4) containing 5 mM MgCl_2_, with or without 50 µg/ml DNase I (Sigma), and incubated at 37°C for 1 h. The MgCl_2_ is required for DNase activity. After incubation, cells were again washed three times in PBS and resuspended at OD_600_ of 0.05 in the buffer used for the subsequent experiment (see below).

### Bacterial adhesion assay

To study bacterial adhesion under flow, glass cover slips, cleaned by dipping them sequentially in acetone, distilled water, ethanol, and distilled water for 1 minute each, were mounted on 3 channel flowcells, each channel measures (length×width×height) 40×4×4 mm (Denmark Technical University, Copenhagen, Denmark). Adhesion of untreated *S. xylosus* was compared to adhesion of *S. xylosus* that was continuously treated with DNase (50 µg/ml), or had temporarily been treated with DNase (50 µg/ml) for 1 h, washed and resuspended in PBS with 5 mM MgCl_2_. All the three suspensions were adjusted to OD_600_ of 0.05 and passed through flowcells (flow rate ∼1 ml per minute), and cells attached to the glass surface were enumerated by phase contrast microscopy after 30, 60 and 90 min.

Continuously shaking batch bacterial adhesion assays were performed in the presence or absence of DNase during the incubation of glass cover slips in bacterial suspensions for 100 min at room temperature with shaking at 120 rpm. The assay was performed on three replicate surfaces incubated individually in bacterial suspensions prepared from the same overnight culture and separated into aliquots before resuspending buffers with or without DNase. Buffers were prepared with three different ionic strengths: a) PBS with 5 mM MgCl_2_ and 30 g l^−1^ NaCl (*I* = 0.70 M) (pH 6.9), b) PBS with 5 mM MgCl_2_ (I = 0.19 M) (pH 7.0), and c) 5 mM MgCl_2_ in deionized water (*I* = 0.015M) (pH 6.1). MgCl_2_ was required for DNase activity. Glass cover slips were recovered and non-adhered bacteria were gently removed by dipping the slides in sterile PBS three times. The remaining bacteria were stained with 10 µl 20x SYBR Green II RNA stain (Sigma-Aldrich), placed on a glass slide and sealed with nail polish to avoid evaporation. Slides were kept in the dark at 4°C until quantification of adhered bacteria by epifluorescence microscopy (Zeiss Axiovert 200M, Carl Zeiss GmgH, Jena, Germany) using Zeiss filterset 10 and 40x or 63x oil immersion objectives. Cells were counted in 190 µm^2^ or 120 µm^2^ grids (depending on the magnification used) at random positions on the slide until a minimum of 1000 cells had been counted. Loosely adhered cells will be removed from the surfaces in the static assay, as large sheer forces are applied when passing the samples through an air/water interfaces. The assay therefore only enumerates the strongly adhered cells, which some studies define as “bacterial retention”. For ease of language, we will refer to both assays as bacterial adhesion in the present study.

### Surface preparation and modification

Glass surfaces with different chemistries were prepared by surface assembly of silanes on square glass cover slips (12×12 mm). Prior to all surface chemical modifications, the cover slips were cleaned by submersing them in Piranha reagent (Ammonium hydroxide (25%), Hydrogen peroxide (35%) and water at 1∶1∶4 volume/volume) for 5 minutes, rinsed with water and dried using jet of nitrogen [53]. Hydrophobic fluoro silane (-CF_3_) functionalised surfaces were prepared by incubation in 100 mM Trimethoxy trifluoropropyl silane in toluene for 12 h and subsequently rinsed by sonication in toluene using a bath sonicator for 1 h, and dried using a jet of nitrogen. Negatively charged carboxyl (-COO^−^) functionalised surfaces were prepared by incubation in 10% volume/volume 3-(Triethoxysilyl) propylsuccinic anhydride in toluene for 15 h. Positively charged amine (-NH_3_^+^) functionalised surfaces were prepared by incubating carboxyl terminated surfaces in 3% volume/volume polyethylene imine (PEI) in water for 12 h. The reaction was catalyzed by NHS-EDC by incubating the carboxyl terminated surfaces with 3 mg of NHS and 30 mg of EDC in 30 ml water prior to incubation with PEI solution. Both the surfaces were then rinsed by sonication in water using a bath sonicator for 1 h and dried using a jet of nitrogen. Piranha treated glass surfaces were used as reference in all the experiments. All surfaces were freshly prepared and used within 24 h.

### Characterization of surfaces

The surface chemical composition of modified surfaces and the unmodified glass was characterized by X-ray photoelectron spectroscopy (XPS). XPS spectra were recorded using a Kratos Axis Ultra^DLD^ instrument (Kratos Ltd, Telford, UK) equipped with a monochromated aluminum anode (Al kα 1486 eV) operating at 150 W (15 kV and 10 mA) with pass energies of 20 eV and 160 eV for high resolution and survey spectra respectively. The charge neutralizer was used to neutralize any positive charge developed during the measurements on electrically non-conducting surfaces. A hybrid lens mode was employed during analysis (electrostatic and magnetic). XPS spectra of the surfaces were recorded at three different spots on each sample. Relative atomic percentages were calculated from the averages of three survey spectra recorded for each sample on three different spot. The take-off angle with respect to normal to the surface was 0° for all measurements. The measured binding energy positions were charge corrected with reference to 285.0 eV, corresponding to the C-C/C-H species. Quantification was conducted using CasaXPS software. A linear background was used to analyze all spectra.

Surface zeta potential measurements were carried out using the surface zeta potential cell in zetasizer nano ZS (Malvern Instruments limited), as described previously [54]. Polystyrene nanoparticles (∼100 nm -PS100 sulphate modified Invitrogen DK) were used as tracer particles. Measurements were made at 125, 250, 375, and 500 µm from the sample surface. An additional measurement was made at 1000 µm to determine the zeta potential of the tracer particles. Surface zetapotentials were calculated from three replicate measurements and expressed as millivolts (mV). Surface zetapotential uncertainties calculated by the software from three measurements were considered as standard deviation.

### Characterisation of bacterial cell surface

Static contact angles were measured on the different surfaces and bacterial lawns using water. Bacterial lawns were prepared as described previously [55]. Briefly, 10^9^ cells per ml of planktonic *S. xylosus* cells from an overnight culture were harvested and treated with DNase or PBS (control) as described above. After treatment, the cells were washed 3 times and resuspended in distilled water, before depositing the suspension on to a cellulose acetate filter membrane containing 0.45 µm diameter pores under negative pressure. All images of liquid drops on surfaces were recorded using a KRUSS DSA100 (KRUSS GmbH, Hamburg, Germany) drop shape analysis system, followed by drop shape analysis using ImageJ software. Water contact angle measurements were done on at least 3 different places on triplicate samples of each surface and averaged.

The zeta potential of bacterial cells was also measured to investigate changes in cell surface charge after removal of eDNA. *S. xylosus* (+/– DNase treatment) were washed and resuspended to OD_600_ of 1.0 in PBS. The conformation of the eDNA on cell surface, and the Zeta potential can be affected by the ionic strength of the buffer. PBS has a relatively high ionic strength. In order to account for the effect of ionic strength on zeta potential, we made the measurements in PBS with two different ionic strengths, 1 mM (I = ∼0.019) and 10 mM (I = 0.19). Triplicate values of the zeta potential were measured for each sample in a zetasizer (Zetasizer nano, Malvern Instruments, UK) at 25°C. Each replicate value was an average of 10 measurements.

### Statistical analysis

Bacterial adhesion was studied on three replicate surfaces incubated individually in bacterial suspensions that were prepared from the same overnight culture, and then separated into aliquots that were either suspended in PBS+MgCl_2_ with and without DNase before submerging samples (one sampler per aliquot). Statistical analyses were done by student t-test and two-way analysis of variance (ANOVA) using R, the free software for statistical computing.

## Supporting Information

Figure S1 **XPS analysis of surfaces with different chemistries.** A: Wide scan spectrum of Piranha-treated glass; B: Carboxyl-functionalised glass. The high resolution C 1s spectrum shows the carboxyl peak at B.E. 289.1 eV; C: Amine-functionalised glass. The wide scan spectrum shows the nitrogen (N 1s) peak at B.E. 397 eV; D: Fluoro-functionalised glass. The wide scan spectrum shows fluorine (F 1s) peak at B.E. 686 eV.
(TIF)
