# Supplementary figures and images for: Surface Physicochemistry and Ionic Strength Affects eDNA’s Role in Bacterial Adhesion to Abiotic Surfaces
Source: PLoS One. 2014 Aug 14;9(8):e105033. doi: 10.1371/journal.pone.0105033 (PMC4133339; doi:10.1371/journal.pone.0105033)

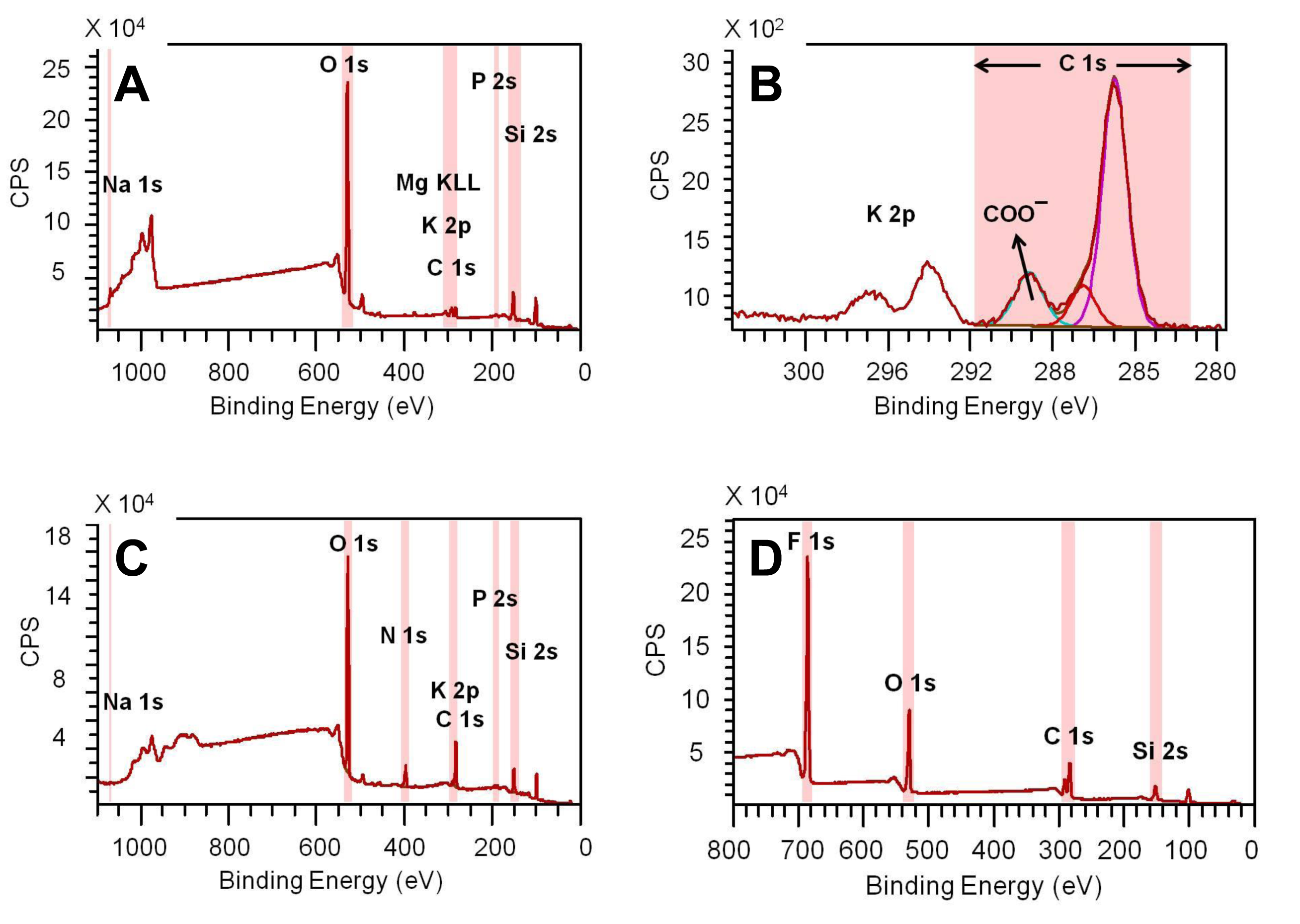

Supplement: Figure S1 — XPS analysis of surfaces with different chemistries. A: Wide scan spectrum of Piranha-treated glass; B: Carboxyl-functionalised glass. The high resolution C 1s spectrum shows the carboxyl peak at B.E. 289.1 eV; C: Amine-functionalised glass. The wide scan spectrum shows the nitrogen (N 1s) peak at B.E. 397 eV; D: Fluoro-functionalised glass. The wide scan spectrum shows fluorine (F 1s) peak at B.E. 686 eV. (TIF) [file pone.0105033.s001.tif]
